# Exploring the Association between Parental Factors and Age of Onset of Alcohol and Tobacco’s Using

**Published:** 2018-10

**Authors:** Zhuo Li TAO, Yi LIU

**Affiliations:** 1. Dept. of Medical Humanities, School of Humanities, Southeast University, Nanjing, China; 2. Health Education Institute, Yunnan Health Department, Kunming, China

**Keywords:** Onset age, Substance use, China

## Abstract

**Background:**

There are few reports about relationship between parents’ age of onset of substance use and their children’s age of onset. The aim of this study was to explore the association between parents’ age of onset of substance use and that of their children, the influence of parents’ factor on their children’s age of onset of substance use.

**Methods:**

Overall, 2036 Chinese college students from ten universities in five cities were assessed for their alcohol and tobacco’s use, and they reported on their parents’ alcohol and tobacco’s use, their parents’ attitudes towards their substance use and their parental education level. Among current substance users, multiple regression analyses were used to test whether their parents’ age of onset of substance use and their parents’ attitudes towards their substance use predict their age of onset of substance use.

**Results:**

Parents of children who used substances earlier showed significantly more tolerance towards their children’s substance use than parents of children with a later onset of substance use. Among current substance users, their parents’ age of onset of substance significantly predicted children’s age of onset. The parents’ attitudes had a significant impact on their children’s age of onset of substance use.

**Conclusion:**

Parental factors (attitudes towards their children’s substance use and parents’ age of onset of substance use) are related to age of onset and substance use in children. Prevention program for parents is necessary, especially for those parents who have a tolerant attitude towards their children’s substance use.

## Introduction

In China, there are 0.35 billion people who use tobacco, and many of them are young people (1). A national survey in China showed that recent tobacco use rate was 13.8% (2). In many societies and cultures, consumption of alcohol during teenage is popular phenomenon (3). The current drinking rate for Chinese youth males was 36.4% and females was 23.8%; the heavy drinking rate for males was 3.3% and females was 1.2% (2).

The earlier the onset of drinking, the more problematic the subsequent use of alcohol (4). The earlier the age of onset of substance use, the easier it was to later develop dependence (5).

“Predictors of alcohol by adolescents have indicated that early age of use is important precursor to later misuse” (6). The association between severity and early onset of substance use among Chinese students was explored (5).

Family risk factors are considered important predictor of misuse of alcohol by adolescents (7). Family history of substance use was correlated with heavier use of alcohol (8) and tobacco (9).

Parental factor was associated with onset and levels of alcohol use in adolescence (10). Higher levels of alcohol use by parents foretell same problems by children (11). Children with drinking parents consider drinking as less harmful and tender to begin drinking earlier (7). Smoking rates of Chinese youths with smoking parents were significantly higher than those youths with no smoking parents (12). Parents permit their children to use substance was considered risk factor for alcohol misuse (13).

We aimed to explore the association between parents’ age of onset of substance use and that of their children, the influence of parents factor on their children’s age of onset of substance use.

## Methods

This survey was approved by Research Committee of School of Social Development and Public Policy, Fudan University in Shanghai.

A cohort of 2200 Chinese college students from ten universities randomly received test battery. One hundred test batteries were not returned and 64 test batteries were not completely filled out and were excluded from analyses. In all 2036 test batteries were useable. The response rate was 92.5%. Out of the participants, there were 1305 females (64.1% of respondents) and 731 males (35.9% of respondents). Mean age of female students was 20.5 yr (SD=1.8), and it of male students was 20.9 yr (SD=2.6). Authorities, lecturers, and students had previously received a written description of the survey.

The test takers were informed that participation was voluntary and anonymous. Students responded to self-administered questionnaires in classroom during a 20 to 30 min session.

### Assessments Questionnaires Alcohol Use Disorders Identification Test (AUDIT)

AUDIT “was developed by WHO as method of screening for excessive alcohol use” (14). It has been found to provide accurate measure of risk across gender, age and culture (15). It contains 10 items about recent alcohol use, alcohol dependence symptoms and alcohol-related problems (16). Its reliability and validity have been shown in studies conducted in different countries (14). In this survey, the Cronbach’s alpha for female participants was 0.87, for male participants was 0.89. In this survey, the scores on the AUDIT were used to indicate the participant’s current alcohol use.

### Fagerstrom Test for Nicotine Dependence (FTND)

FTND was used for assessing nicotine dependence and consists of eight items. It has shown acceptable internal consistency and reliability (16). In this survey, the Cronbach’s alpha for female participants was 0.87, for male participants was 0.88. In this survey, the scores on FTND were used to indicate participant’s current tobacco use.

### Questions about age of onset of alcohol use

The construct was measured by one item assessing participant’s age of onset of drinking. Age at first use of alcohol was defined as follows: “Not counting sips, how old were you the very first time you had a drink of beer, wine (or rice wine), or liquor?” (3).

Ages of onset was classified as code 6, which stood for “younger than 11 yr”, code 5 for “11 to 12 yr”, code 4 for “13 to 14 yr”, code 3 for “15 to 16 yr”, code 2 for “17 to 18 yr” or code 1 for “older than 18 yr”.

To study the influence of parents’ attitudes on their children’s age of onset of alcohol use, participants were classified into three groups (age of onset 13 yr and earlier), (age of onset 14 yr to 17 yr), and (age of onset 18 yr and later) according to their age of onset of alcohol. Earlier onset group (age of onset 13 yr and earlier) (140 males and 91females) was compared with control group (age of onset 18 yr and later) (68 males, 139 females).

Participants also provided information about age of onset of alcohol use of their parents’ using, the same question.

### Questions about age of onset tobacco use

Participants filled one item question about age of onset of their own tobacco use. The item was: “When did you begin smoking?” (Smoke a whole cigarette), the age of onset was classified as the age of onset of alcohol use.

To study influence of parents’ attitudes on their children’s age of onset of tobacco use, three groups were created like alcohol use groups.

### Questions about parents’ attitudes towards their children’s alcohol use

The construct was measured by one item assessing their parents’ attitudes towards their children’s alcohol use. The item was, “what are your parents’ attitudes towards your alcohol use?” The response options were “Support” or “Not support”.

### Questions about parents’ attitudes towards their children’s tobacco use.

The participants filled one item question about their parents’ attitudes towards their alcohol use. The question and responses were the same as parents’ attitudes towards their children’s alcohol use.

### Parent alcohol use during last 12 months

Participants filled one item questions about how frequently their parents’ drinking alcohol during last 12 months. The question was adapted from (14), which was, “How often does he or she have a drink containing alcohol?” The participants answered the question separately for their father and mother.

### Parent tobacco use during last 12 months

Participants filled one item question about how frequently their parents’ using tobacco during last 12 months.

Response options were: “no”, “10 or less/day”, “21–30/day”, and “31or more/day”. The question was adapted from (16). Participants answered the question separately for their father and mother.

### Parental education level

This construct was measured by one item asking education level of parents. Participants were classified as code 1, which stood for “junior high school or lower”, code 2 for “senior high school”, code 3 for “bachelor’s degree” or code 4 for “master’s degree or higher”. Participants answered the question separately for their father and mother.

### Data Analysis

Analyses were conducted in SPSS 14.0 (Chicago, IL, USA). Chi-square tests were used to compare differences between early age of onset group and control group on their parents’ attitudes towards their substance use.

Among current substance users, hierarchical regression analyses were conducted to assess relationship between children’s age of onset of tobacco and alcohol’s use and influential factors (parents’ age of onset of tobacco and alcohol’s use, parent attitude towards their children tobacco and alcohol’s use). Path analysis was used to describe relationship between influential factors (parents’ age of onset of tobacco and alcohol’s use, parent attitude towards their children’s tobacco and alcohol’s use), children’s age of onset of substance use and children’s current substance use (scores on AUDIT and FTND). Statistical significance was based on two-sided tests evaluated at the 0.05 significance level.

## Results

Among all participants, there were 108 male (14.8% of all male participants) who reported currently using tobacco, 384 males (52.5% of all males) and 270 females (20.7% of all females) who currently consumed alcohol.

Among current tobacco users, parents of female current tobacco users showed significantly earlier age of onset of tobacco use than those of male tobacco users (Table 1).

**Table 1:** Comparison between male and female current tobacco users

	***Male***	***Female***	***F***	***P***
N	108	19		
	M (SD)	M (SD)		
Age(yr)	21.73 (4.77)	20.61 (1.33)	0.49	ns
Father education level	1.79 (0.76)	1.94 (0.99)	3.34	ns
Mother education level	1.51 (0.81)	1.81 (0.91)	0.06	ns
Children onset age of tobacco use	4.70 (2.99)	6.06 (2.79)	1.69	ns
Fragestrome score of children	4.75 (2.09)	4.26 (1.52)	2.83	ns
Parents attitudes toward children’s tobacco use	0.65 (0.48)	0.64 (0.50)	0.02	ns
Father onset age of tobacco use	1.17 (1.29)	2.88 (3.56)	24.62	<0.01
Mother onset age of tobacco use	0.22 (0.94)	2.00 (3.02)	22.18	<0.01

Among current alcohol users, fathers of females had significantly higher education level than fathers of male current alcohol users. Fathers of male current alcohol users had significantly earlier onset of alcohol use than those of female participants (Table 2).

**Table 2:** Comparison between male and female current alcohol users

	***Male***	***Female***	***F***	***P***
N	384	270		
	M(SD)	M(SD)		
Age(yr)	20.90 (3.11)	20.45(1.91)	3.52	ns
Father education level	1.92 (0.85)	2.07(0.98)	7.29	<0.01
Mother education level	1.73 (0.85)	1.93 (0.90)	0.22	ns
Children onset age of alcohol use	5.01(2.61)	4.22 (2.76)	1.03	ns
AUDIT scores of children	4.84 (1.95)	4.29 (1.49)	1.46	ns
Parents attitudes toward children’s alcohol use	0.86 (0.34)	0.88(0.33)	0.58	ns
Father onset age of alcohol use	3.01(2.83)	2.64(2.44)	4.92	<0.05
Mother onset age of alcohol use	1.24 (2.24)	1.59 (2.50)	0.64	ns

The data in table 1 and 2 were already coded

Among current substance users, multivariable regression was used to detect predictor of children’s age of onset of tobacco and alcohol’s use. Gender, age, father’s and mother’s education level served as control variables, Children’s age of onset of tobacco and alcohol’s use served as dependent variables. The following variables served as independent variables: parents’ attitudes towards children’s tobacco and alcohol’s use, father’s and mother’s age of onset of tobacco and alcohol’s use. As Table 3 shows, after controlling for gender, age and father’s and mother’s education level, the independent factors (parents’ attitudes towards children’s tobacco use, father’s and mother’s age of onset of tobacco use) were entered into the regression block, and parents’ attitudes significantly predicted children’s age of onset of tobacco use (F=4.46, OR=2.14, 95%CI0.66–2.94, R^2^ change =0.12, *P*<0.01). This finding supported the second hypothesis.

**Table 3:** Hierarchical regression analyses for relationship between parent influence factors and children’s onset age of tobacco use

**Dependent Variable and Predictors**	***First step***	***Second step***	***Third step***	***Fourth step***
Gender	0.42	0.43	0.19	0.38
Age (yr)	0.29	0.38	0.33	0.39
Father education level	−0.97	−0.71	−0.69	−0.73
Mother education level	1.00	0.96	0.92	0.75
Parents attitudes toward children’s tobacco use		2.14^**^	1.79^**^	1.78^**^
Father onset age of tobacco use			0.32^**^	
Mother onset age of tobacco use				0.26^*^
R^2^	0.08	0.20	0.28	0.28
R^2^ change	0.08	0.12	0.08	0.05
F change	2.01	13.18	10.30	6.29

** *P*<0.01

* *P*<0.05

Father’s and mother’s age of onset of tobacco use were highly correlated (r=0.72, *P*<0.01), and multicol-linearity was considered a substantial problem. Therefore, father’s and mother’s age of onset of tobacco use were entered the regression block separately. Father’s age of onset of tobacco use significantly predicted children’s age of onset (F=5.83, OR=0.30, 95%CI 0.12–0.54, R^2^ change =0.08, *P*<0.01). Mother’s age of onset of tobacco use significantly predicted children’s age of onset (F=5.77, OR=0.25, 95%CI0.05–0.47, R^2^ change =0.05, *P*<0.05). These results supported the first hypothesis.

Above and beyond control factors, the independent factors (parents’ attitudes towards children’s alcohol use, father’s and mother’s age of onset of alcohol use) were entered into the regression block (Table 4). Parents attitudes significantly predicted their children’s age of onset of alcohol use (F=4.01, OR=1.76, 95%CI0.77–2.74, R^2^ change =0.06, *P*<0.01), which supported the second hypothesis.

**Table 4:** Hierarchical regression analyses for relationship between parent influence factors and children’s onset age of alcohol use

**Dependent Variable and Predictors**	***First step***	***Second step***	***Third step***	***Fourth step***
Gender	−0.83	−0.93	−0.83	−0.87
Age (yr)	−0.14	−0.10	−0.14	−0.11
Father education level	−0.15	−0.06	0.05	0.01
Mother education level	0.26	0.20	0.07	−0.02
Parents attitudes toward children’s alcohol use		2.05^**^	1.91^**^	1.55^**^
Father onset age of alcohol use			0.25^**^	
Mother onset age of alcohol use				0.39^**^
R^2^	0.03	0.09	0.15	0.18
R^2^ change	0.03	0.06	0.06	0.10
F change	1.32	14.44	14.15	23.58

** *P*<0.01 //

* *P*<0.05

Father’s and mother’s age of onset of alcohol use were highly correlated (r=0.54, *P*<0.01), and multicollinearity was considered a substantial problem. Therefore, father’s and mother’s age of onset of alcohol use were entered into the regression block separately. Father’s age of onset of alcohol use significantly predicted children’s age of onset (F=5.92, OR=0.25, 95%CI0.12–0.38, *P*<0.01), and accounted for 6.0% of the adjusted variance. This finding supported the first hypothesis. Mother’s age of onset of alcohol use significantly predicted children’s age of onset (F=7.29, OR=0.39, 95%CI0.23–0.55, *P*<0.01) and accounted for 10.0% of the adjusted variance. This finding also supported the first hypothesis.

We calculated correlations between father’s and mother’s age of onset of tobacco use and their children’s scores on FTND (their current tobacco use) and father’s and mother’s age of onset of alcohol use and their children’s scores on AUDIT (their current alcohol use). None of the correlations were significant.

Father’s age of onset of tobacco use significantly predicted their children’s age of onset (β=0.30, *P*<0.01), parents’ attitude towards their children’s tobacco use significantly predicted children’s age of onset of tobacco use (β=0.30, *P*<0.01), and children’s age of onset of tobacco use significantly predicted scores on FTND (their current tobacco use) (β=0.29, *P*<0.05).

Mother’s age of onset of tobacco use significantly predicted their children’s age of onset (β=0.25, *P*<0.01), parents’ attitudes towards their children’s tobacco use significantly predicted children’s age of onset of tobacco use (β=0.30, *P*<0.01), and children’s age of onset of tobacco use significantly predicted their FTND scores (their current tobacco use) (β=0.29, *P*<0.05). Father’s and mother’s age of onset of tobacco use had an indirect impact on their children’s current tobacco use through their children’s age of onset of tobacco use, which supported the third hypothesis.

Among all participants, significantly fewer parents of males in earlier alcohol onset group (13 yr or younger) were against their sons’ alcohol use compared to parents of males in control group (18 yr or later) (7.1%) vs (29.4%); χ^2^ =31.38, df=16, *P*<0.05). Similarly, significantly fewer parents of females in earlier alcohol onset group (13 yr or younger) were against their daughters’ alcohol use compared to parents of females in control group (18 yr or later) ((7.7%) vs (24.5%); χ^2^ =34.48, df=14, *P*<0.01).

Compared to parents of males in control group (18 yr or later), parents of males in earlier alcohol onset group (13 yr or earlier) began to consume alcohol significantly earlier (*P*<0.001). Compared to parents of males in control group (18 yr or later), parents of males in earlier tobacco use onset group (13 yr or earlier) began to consume tobacco significantly earlier (*P*<0.001). Compared to parents of females in control group (18 yr or later), parents of females in earlier tobacco use onset group (13 yr or earlier) began to consume tobacco significantly earlier (*P*<0.001).

## Discussion

The major finding in this survey was based on report of a group of Chinese college students, and it showed that their parents' age of onset of tobacco and alcohol’s use predicted that of these students’ age of onset. The results supported the findings that found parenting factors were associated with the onset of drinking in adolescence (11). In comparison to genetic factors, environmental factors explained more variance in the initiation of alcohol use (17). Parents’ attitudes about their children’s tobacco and alcohol’s use were related to their children’s age of onset, and significantly fewer parents were against their sons’ alcohol use in earlier alcohol use onset group (13 yr or younger) compared to control group. Additionally, parents’ attitudes toward their children’s tobacco and alcohol’s use significantly impacted their children’s age of onset of alcohol and tobacco’s use. It should be interesting to discuss why parents with children with an earlier age of onset of substance use were more tolerant towards their children’s substance use. It is possible that parents of children with earlier age of onset of substance use who began to use substance earlier, their attitudes towards their children are a rationalization, a self-defence mechanism in line with Freud’s theory. Parents showed tolerance towards their children's behavior, in essence, tolerance of their own behavior. According to cognitive dissonance theory (18), if parents asked their children to cease substance use, but parents themselves could not do it, then parents would feel cognitive dissonance, which could cause them discomfort. For example, in China, most tobacco users do not want to cease tobacco use, with 39.1% of tobacco user not want to cease use and 56.8% of tobacco users not want to make a decision. Only 4.1% want to stop their tobacco use (12), and these smoking parents must want to change their cognitive dissonance in order to pass their behaviours.

There are few reports of whether father’s or mother’s substance use has a stronger relationship with their children’s substance use.

We found that fathers’ age of onset had a stronger association with their children’s age of onset of their tobacco use than mothers’ age of onset among Chinese participants. In China, fathers’ tobacco use had more influence on their children. There were significant gender differences among Chinese students concerning substance use. Substance use (alcohol and tobacco’s use) was greatest among adult Chinese males. A study in China with 16407 participants showed that smoking rates among people age 15 yr and over were 66.0% for males and 3.1% for females (19). A study with 23513 Chinese participants showed that 84% of males and 30% of females drank alcohol, and 16% of males and 2.5% of females drank alcohol every day (20). This finding has also been reported in other countries. There are clear gender differences in tobacco use, and more men than women use tobacco products (21). Men generally drink more than women, and men and women report different needs, reasons and motivations for drinking (22). In Asia and other Eastern countries, society is more tolerant towards men using tobacco than women (23).

More research is needed on why mothers’ age of onset of alcohol use had stronger impact on children’s age of onset than did father’s age of onset of alcohol use.

First limitations of this survey: age of onset for substance use by parents’ was reported by their children, not by parents themselves. The results should be carefully interpreted.

Second, the article is based on subjective self-report survey, and for the sake of validity, further studies are needed to replicate the findings.

## Conclusion

This study raises important policy implications for the development of alcohol and tobacco prevention programmes for youths. The survey showed that the parents’ attitudes towards their children’s substance use have a positive impact on their children’s substance use, such that the prevention programmes for parents are also necessary, especially for parents who use substances. The parents who started using substances earlier were more tolerant towards their children’s substance use, and their children started using substances than the other children. In this survey, we also found that the children who started tobacco earlier consumed tobacco more heavily now. This pathway should be interrupted.

## Ethical considerations

Ethical issues (Including plagiarism, informed consent, misconduct, data fabrication and/or falsification, double publication and/or submission, redundancy, etc.) have been completely observed by the authors.
